# Differentiating Creatine and Phosphocreatine In Vivo Using 3 T ^1^H MR Spectroscopy

**DOI:** 10.1002/mrm.70171

**Published:** 2025-11-09

**Authors:** Ralph E. Hurd, Meng Gu, Philip M. Adamson, Kenichi Okamura, Masafumi Shibata, Yoshikazu Ono, Moussa Haidar, Kirk Riemer, Frank L. Hanley, Daniel M. Spielman

**Affiliations:** ^1^ Department of Radiology Stanford University School of Medicine Stanford California USA; ^2^ Department of Electrical Engineering Stanford University School of Medicine Stanford California USA; ^3^ Department of Cardiothoracic Surgery Stanford University School of Medicine Stanford California USA

**Keywords:** circulatory arrest, creatine, macromolecules, MR spectroscopy, non‐metabolites, phosphocreatine, synthetic MRS

## Abstract

**Purpose:**

Validation and improvements to the proton MR spectroscopic measurement of creatine and phosphocreatine in vivo at 3 T.

**Methods:**

Spectral simulations were used to evaluate and explore the mitigation of LCModel fitting bias due to lineshape, signal‐to‐noise (SNR) and non‐metabolite backgrounds with a focus on phosphocreatine and the spline regularization settings in the fitting model.

**Results:**

Although phosphocreatine and creatine are visually unresolved, excellent fitting estimates are achieved in the absence of non‐metabolite background using default settings. However, in the presence of non‐metabolite background, bias is observed and depends on lineshape, SNR and differences in the non‐metabolite background. A more flexible setting for the LCModel spline largely mitigates this bias, allowing for the confidence to use proton MRS for PCr dynamic studies.

**Conclusion:**

Without prior knowledge of the non‐metabolite signal, default settings of LCModel can introduce significant bias. Modified setup for LCModel improves quantitation of phosphocreatine and reduces bias in the overall set of metabolites.

## 1 Introduction

^1^H MR spectra of phosphocreatine (PCr) and creatine (Cre) are almost identical and remain visually unresolved for in vivo data with few exceptions [1, 2]. Homogeneity is rarely sufficient to resolve the small separation between PCr and Cre. Independent of field strength, the full width at half maximum (FWHM) linewidth must be less than the peak separation in ppm to visually resolve those peaks. For that reason, the combined total creatine tCr (PCr + Cre) is reported most frequently. Despite these limitations, in an in vivo study of cardiopulmonary bypass (CPB) in piglets at 3T [3, 4], we found that using the temperature‐dependent chemical shifts, routine linear least squares fitting of PCr gave results that were qualitatively consistent with different bypass conditions. Specifically, the reduction of PCr during circulatory arrest. These relative PCr measurements have been very useful for comparison of bypass methods and surgical conditions [5], but have not been fully validated. In addition, rapid and complete depletion of PCr during a 37°C circulatory arrest with slow recovery after reperfusion, compared with slow incomplete loss of PCr during an 18°C circulatory arrest with fast recovery during reperfusion and rewarming suggests that PCr kinetics (a physiologically important measurement) may be accessible using proton MRS. In this study, we use simulations based on 3 T MRS of normal humans and piglet bypass studies to validate and quantify biases in ^1^H MR spectroscopy. Bias mitigation is focused on using LCModel [6, 7] with modified control parameters such as dknmtn which impacts the baseline spline regularization algorithm. A matrix of simulations covering SNR from 10 to 100, FWHM linewidths of 0.02–0.07 ppm, and native plus alternate backgrounds, was used to optimize the LCModel setup.

## 2 Methods

### 2.1 Human Data

A MR spectrum from a 15 cc ROI prescribed to include the anterior cingulate cortex (ACC) in a normal human subject was chosen for simulation input. The initial spectra for simulation are chosen for quality including high SNR, good spectral resolution and minimal visual baseline artifacts. As such, these spectra are assumed to provide a good starting point to establish a realistic ground truth set of metabolites and a minimally challenging native non‐metabolite baseline.

### 2.2 Piglet Data

Two studies, deep hypothermia circulatory arrest (DHCA) at a nominal circulatory arrest (CA) temperature of 18°C, and CA at 37°C were chosen from previous archived studies [8, 9] for simulation and validation. All animal procedures were conducted under an approved Stanford University Institutional Animal Care and Use Committee protocol. After median sternotomy and central cannulation, newborn pigs (2 weeks old, 10 and 11 kg) were placed on a GE MR750 3 T scanner (GE Healthcare, Waukesha, WI, USA) with the head placed in an 8‐channel knee coil (37°C CA study) or in a 16‐channel flex RF coil (18°C DHCA study). The total elapsed magnet time, including baseline measurements, CA, and recovery, was 3.5 h/animal. MR imaging at baseline and recovery included anatomical T_1_ and T_2_ weighted scans (T_1_ parameters 3D BRAVO, 450 ms prep, TE/TR: 2.6/6.2 ms, 1 mm slice thickness: 150 locations per slab, 256 × 256 matrix, and 4 min 46 s scan time); (T_2_ parameters: 2D Fast Spin Echo, ETL 12, 13.4 ms echo spacing, TE/TR: 60/2000 ms, 1 mm slice thickness: 72 locations per slab, 256 × 192 matrix, 4 min 1 s scan time). MR spectra were acquired repeatedly during the remainder of the time.

### 2.3 MR Spectra

The human spectrum was taken over a 50 × 30 × 10 mm^3^ mid‐frontal volume centered around the ACC. All piglet spectra are from a 12 × 12 × 15 mm^3^ voxel placed in the right midbrain as previously described [3, 4, 9]. The human spectrum is the sum of a 3 × 5 matrix of 1 cc elements from a focal MRSI (16 × 16, FOV 160, NEX 1) scan prescribed to include ACC in a normal healthy volunteer. Multiple subvoxels were summed for SNR. Data were collected on a GE MR750 or a 3 T UHP (GE Healthcare, Waukesha, WI. USA) using sLASER TR 2000, TE 304096 points and 5000 Hz spectral bandwidth (WIP). Additional information is included in supplemental Table S1 in the MRSinMRS format [10]. MRS prescan including center frequency correction and linear shim adjustment was applied for each spectrum. For the single voxel piglet data either 64 or 128 total excitations were collected along with eight water unsuppressed excitations. 5% residual water was retained in the water suppressed data by design, and subsequently removed by pure water subtraction. In addition to the water subtraction, pre‐processing included restricted‐band eddy current correction [11]. Data collected for the DHCA study also included a 15 mm^3^ sensitive point filter (SPF) [9] interleaved in the dynamic series. Both SPF and single voxel (SV) data were included in the analysis. Spectral linewidths ranged from 0.019 to 0.053 ppm in the piglet studies and 0.038 ppm in the human example.

### 2.4 LCModel Control Parameters

LCModel (version 6.3‐1J [6, 7]) was used with standard default conditions, or modified to reduce bias from the underestimation of non‐metabolite contributions. Changes to the LCModel control file are detailed in supplemental Table S2. One key parameter dknmtn impacts the spline estimate of non‐metabolite signal and was set at 0.01 0.02 0.03 0.05 0.075 0.150 (default) and 1.00 to assess impact on both PCr/tCr bias and total bias (sum of absolute values of bias in all metabolites). In addition to bias, LCModel output of FWHM, SNR, knots and the spline regularization coefficients for baseline alphaB and signal alphaS were recorded. Lowering dknmtn from the default of 0.150 decreases the SNR‐dependent smoothing of the non‐metabolite spline estimates applied by the regularization process (alphaB) and for FWHM less than 0.058 ppm lowering dknmtn can increase the number of knots [6, 12]. Knot spacing is limited to rho*FWHM where rho is set to 2.6. The rho*FWHM limit helps enforce the assumption that the regularization of metabolite and non‐metabolite signal is separable [12]. Increase of dknmtn above the default of 0.150 ppm reduces the number of spline knots yielding the more constrained spline explored previously [13, 14]. Reduction of dknmtn below the default decreases the regularization coefficient alphaB reducing spline smoothing. In an early study dknmtn = 0.05 was shown to provide a fairly good estimate of macromolecule contributions without any added prior knowledge [2]. One limitation appeared to be that smoothing was still too aggressive for the narrowest macromolecule peaks. These encouraging early results led us to explore even lower settings. Other changes of note are reduction of allowed individual shift correction for PCr and Cre (alsdsh reduced from default of 0.004 ppm to 0.001 ppm), and the addition of macromolecule peak at 3.0 ppm (MM30). These minor changes were also included in the dknmtn study matrix.

### 2.5 LCModel Basis Sets

As previously described [4, 9], the basis sets were primarily constructed from experimental 3 T spectra of metabolites at 20°C and 37°C, pH 7.2. Basis sets for the temperatures between 15°C and 37°C used the nearest temperature basis spectra and a linear extrapolation of the creatine and phosphocreatine chemical shift temperature dependence. Experimental PCr and Cre chemical shifts are provided in supplemental Table S3 and Figure S1.

### 2.6 Simulated Spectra

To validate the LCModel estimates of PCr, simulated spectra were constructed with a known metabolite ground truth, adding lineshape, non‐metabolite signal (macromolecules, out‐of‐band, water and baseline distortion), and noise to mimic in vivo results. The basic scheme is shown in supplemental Figure S4. In vivo lineshape, and non‐metabolite contributions vary significantly with temperature, circulatory arrest, and recovery conditions. Circulatory arrest spectral‐baseline can be dominated by increasing levels of deoxy‐Hemoglobin (deoxyHb), which produces a powder pattern from the water in and near vasculature [15, 16]. To aid recovery, the blood supplied by the bypass pump is highly oxygenated, reducing the spectral linewidth in recovery spectra. To get an initial high SNR estimation of metabolites and non‐metabolite signal, spectra collected over 10 min were averaged for baseline, circulatory arrest, and recovery conditions. The human example with a larger ROI had SNR greater than 50 and was sufficient for the initial estimate. LCModel‐computed metabolite levels from these in vivo fits were then adopted as ground truth to construct simulated spectra by combining metabolite basis set spectra. After lineshape and signal of the simulated spectra were matched to the in vivo spectrum, the metabolite‐only simulations were subtracted from the in vivo result to get an experimental non‐metabolite contribution for each of the three distinct conditions. Before the combination of the experimental non‐metabolite with the ground truth metabolite simulation, mild Gaussian apodization (10 Hz) was used to reduce any residual metabolite contamination in the broad non‐metabolite FID. After adding noise to match the high SNR in vivo spectra, LCModel fits of the simulated data were then compared with LCModel fits of the in vivo spectra. Phosphocreatine/total creatine (PCr/tCr) concentration was then adjusted to provide a titration from 0.00 to 1.00 in 0.1 unit steps. For this simulated titration, additional Gaussian noise with a different seed for each simulation was added to match the normal 2‐min spectral SNR provided by LCModel, which is defined as the “ratio of the maximum in the spectrum‐minus‐baseline over the Analysis Window to twice the rms residuals”. In addition to the PCr titrations, simulations were modified by the addition of noise, linewidth and swapping in different non‐metabolite backgrounds. The search for an optimum dknmtn setting included simulations with SNR from 10 to 100, FWHM linewidths from 0.02 to 0.07 ppm, using native non‐metabolite background plus swapping in a range of more challenging non‐metabolite situations. The input for the initial simulation was based on a high‐quality human ACC spectrum. The default settings for LCModel and a largely experimental 23‐metabolite‐only basis set were used to provide the initial metabolite estimates which were then adopted as ground truth for the analysis. The metabolite‐only simulation was broadened by a 1.25 Hz Lorentzian followed by Gaussian apodization to roughly match the in vivo condition. After the extracted non‐metabolite background and Gaussian noise were added, the simulation was validated against the original spectrum by LCModel fit. Linewidths of approximately 0.02, 0.03, 0.04 0.05 and 0.07 ppm were achieved starting with the 1.25 Hz Lorentzian simulation followed by Gaussian apodization of 2, 3, 4, 6, and 8 Hz. An asymmetric lineshape was also simulated by shifting 30% of the signal downfield by 0.02 ppm (asymmetry in excess of the PCr Cre CH_2_ separation). Extracted non‐metabolite contributions (native background, alternate more challenging backgrounds taken from the extreme pig bypass study, plus a simple 25 Hz Gaussian peak placed near the PCr + Cre CH_2_'s) were swapped in to test the impact of dknmtn over a range of SNR, linewidth, and non‐metabolite backgrounds on both PCr/tCr bias and total metabolite bias.

### 2.7 Dynamic Plots

Fits to each piglet spectrum collected during CPB including distinct baseline (PreCA), circulatory arrest (CA) and recovery intervals were plotted versus time relative to the start of CA. Brain temperature for each time point was estimated from water chemical shift relative to N‐acetyl Aspartate NAA [17]. Standard deviations (SD) of metabolites are taken from the LCModel estimation of SD, and a 2‐point running average was applied. Data were converted to millimolar (mM), assuming a creatine concentration of 8.0 mM.

## 3 Results

### 3.1 Simulated Spectra

**FIGURE 1:**
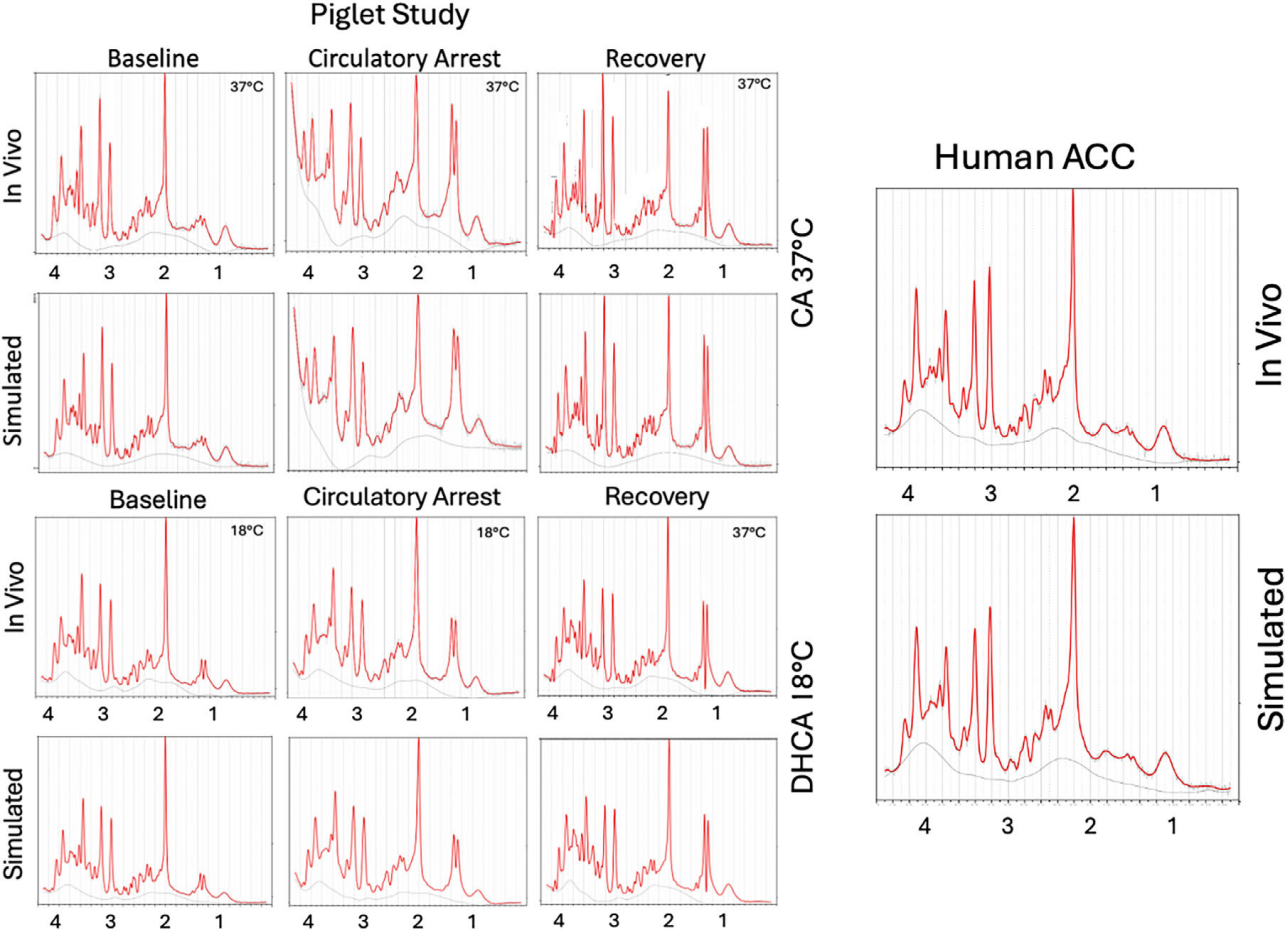
LCModel fits of in vivo and simulated spectra based on a normal human volunteer and on piglet spectra at baseline, circulatory arrest, and recovery from previously described DHCA 18°C and CA 37°C studies. Estimated metabolite ratios from a high SNR average (5 spectra for each interval) were used as the input for the piglet simulations. After addition of linewidth, non‐metabolite signal and noise to the simulations, LCModel estimates showed good agreement between in vivo and simulation. Linewidth and some of the non‐metabolite signal change during circulatory arrest is due to deoxyHb water powder pattern and T2* broadening. Decrease in linewidth during recovery is also a common feature of these spectra, likely due to elevated blood O_2_ used to help with speed of recovery.

Figure 1 shows LCModel fits for in vivo and simulated data. In vivo spectral fits for a normal human example and piglet studies at baseline, circulatory arrest, and recovery periods in a 37°C bypass study and for a 18°C DHCA bypass study are shown along with fits of the ground truth simulations. For these high quality spectra (SNR greater than 30) output levels match the simulated input for all metabolites with minimal bias. To achieve SNR greater than 30 for piglet examples 11–13 min averaged spectra (sum of 3128 average spectra in the 37°C CA study and the sum of 5 64 average spectra for the 18°C DHCA study), were used. Simulations were then modified to contain PCr/tCr ratios from 0 to 1.0 in steps of 0.1. Linewidth, noise and non‐metabolite contributions were then added to explore how well the fitting method estimated the PCr/tCr ratio.

### 3.2 Non‐Metabolite Contributions

**FIGURE 2:**
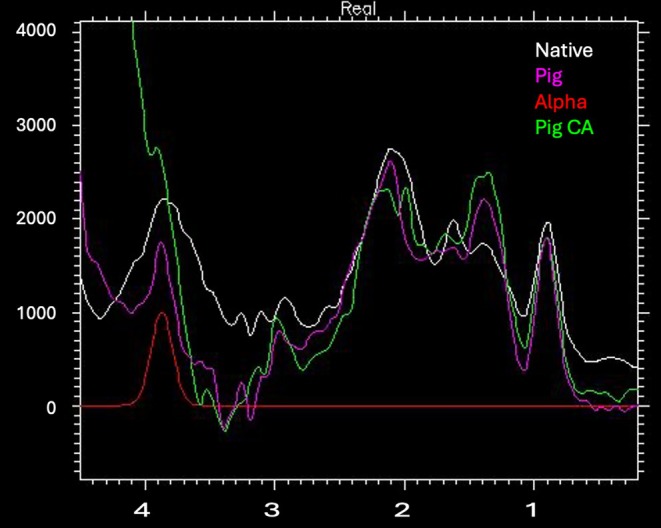
Non‐metabolite contributions. Backgrounds from normal human volunteer (white trace), 37°C piglet preCA interval (purple trace), 37°C piglet CA interval (green trace), and a simple but challenging 25 Hz Gaussian background at 3.85 ppm constructed to represent the most likely source of bias for PCr (red trace). Note the significant variability especially near 3.9 ppm region where baseline estimation error is expected to have biggest effect on the separation and estimation of PCr and Cre.

Figure 2 shows experimental non‐metabolite contributions (difference between metabolite estimates and the in vivo input spectra). Reduced macromolecule contributions at low temperature (not shown) and the water powder pattern from deoxyHb during CA have substantial effects on the overall non‐metabolite signal. The variability of non‐metabolite signal near PCr and Cre methylene signal around 3.9 ppm makes any attempt at prior knowledge solutions a potential problem for the substantially overlapping PCr and Cre resonances. The range of non‐metabolite backgrounds under different temperature and physiological conditions suggest a need for estimates of this broad component without the requirement for over‐constrained prior knowledge and without impact on the estimates of the narrower metabolite signals. To expand on the range of unmodeled non‐metabolite backgrounds a simple but challenging 25 Hz Gaussian at 3.85 ppm was included. This provides a visually easy target to evaluate the spline regularization estimate and any overfitting over the remainder of the baseline.

### 3.3 Bias, Linewidth, dknmtn, and Non‐Metabolite Contributions

**FIGURE 3:**
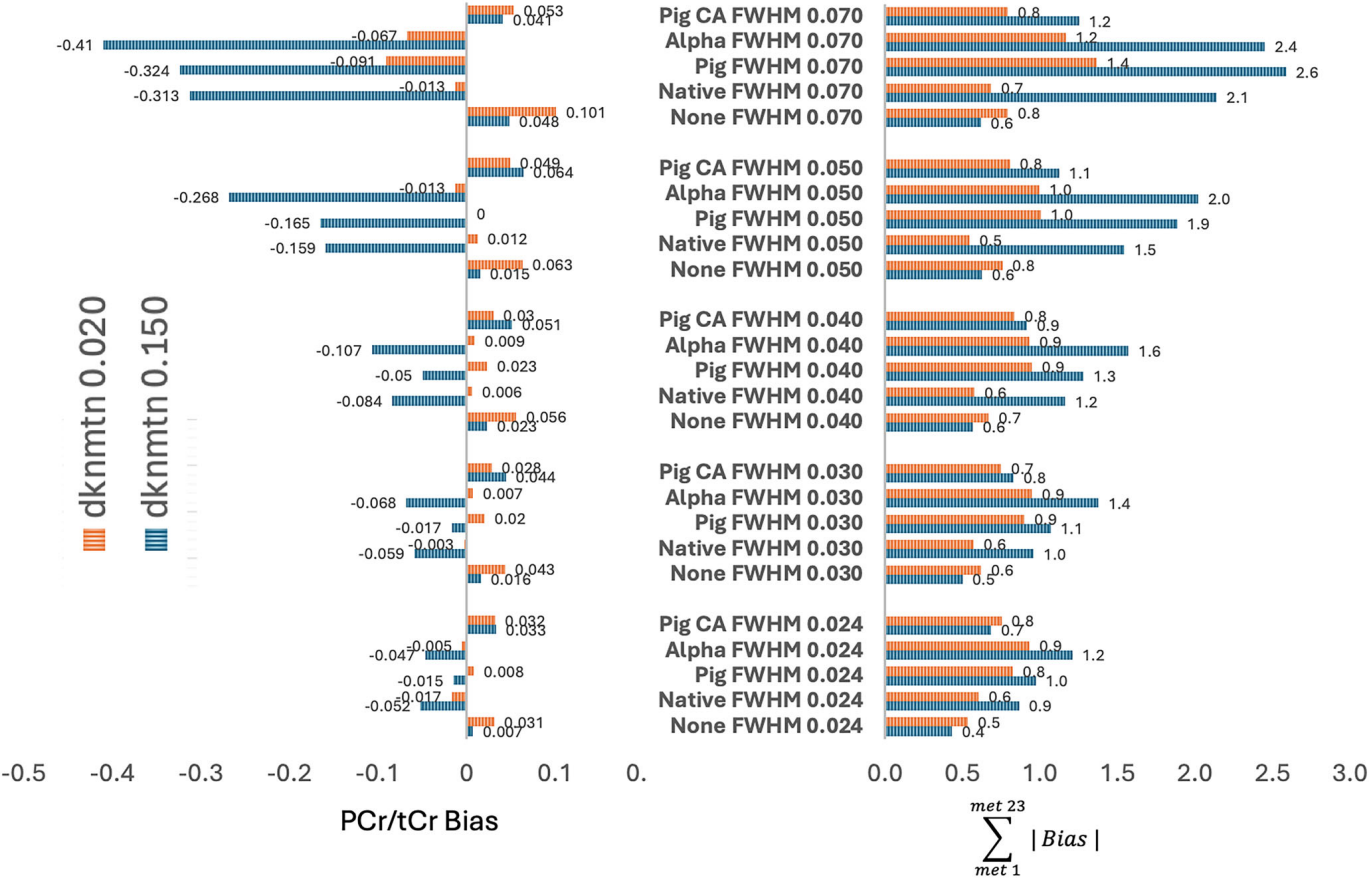
Bar chart illustrating PCr/tCr bias and total metabolite bias comparing dknmtn at 0.020 versus 0.150 (default) over a range of linewidths (0.024–0.70) and backgrounds, swapping in piglet backgrounds, no background and the simple 25 Hz alpha background for the native non‐metabolite background associated with this normal human spectrum. Noise was constant with a nominal value of 25. This figure illustrates linewidth and background dependent bias for the default dknmtn setting (0.150). The highest biases were observed with native, pig and the simple but challenging alpha backgrounds. Bias error was most pronounced at 0.070 ppm linewidth.

Figure 3 shows a bar chart illustrating PCr/tCr bias and total bias as a function of dknmtn setting and linewidth (0.024–0.070 ppm) at the same noise level yielding a nominal SNR of 25 at FWHM 0.040. The default dknmtn setting of 0.150 shows more bias than dknmtn 0.020 with native, pig and alpha backgrounds yielding the most bias. An overall flow chart of the simulation and fit matrices, output and analysis are provided in supplemental Figure S5.

### 3.4 PCr/tCr Bias, SNR, dknmtn, Knots, alphaB and Linewidth

**FIGURE 4:**
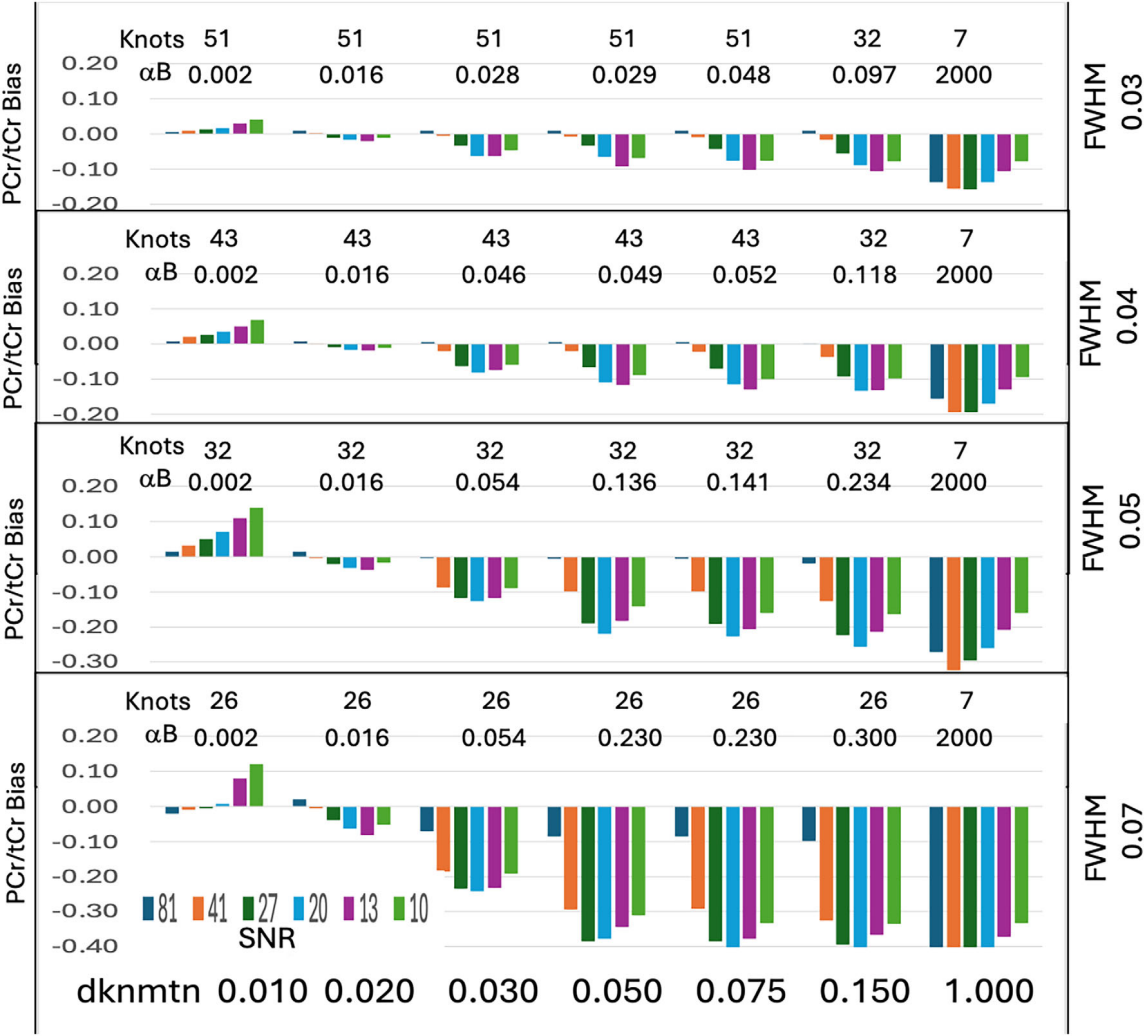
Column chart illustrating PCr/tCr bias over a range SNR, FWHM linewidths and dknmtn settings. In this example, the simple alpha background and normal human simulation was used. The number of knots and the baseline regularization coefficients (alphaB) are listed. At FWHM linewidths of 0.05 or above, the number of knots doesn't change for dknmtn values below default, whereas the baseline regularization coefficients do change. A dknmtn setting of 0.02 was found to be the best value for this metric.

Figure 4 shows a column chart illustrating PCr/tCr bias and total bias for a range of dknmtn values (0.010, 0.020, 0.030, 0.050, 0.075, 0.150, and 1.00), linewidths of 0.030, 0.040, 0.050, and 0.070 ppm and a SNR range of 10–81. In this example ground truth metabolites are from the human simulation and the background was the simple 25 Hz Gaussian. Knots and baseline regularization coefficients are given. At FWHM 0.05 ppm the number of knots are the same for all dknmtn settings lower than default 0.150. At dknmtn 1.0 the number of knots is reduced to 7 for all linewidths tested. The number of knots is as expected for the minimum spacing limit of rho*FWHM where default rho is 2.6 and for a spacing of 1 ppm at dknmtn of 1.00.

### 3.5 PCr/tCr Titrations

**FIGURE 5:**
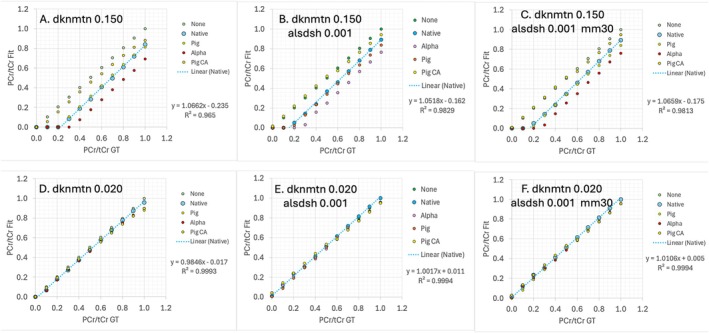
Titrations of PCr/tCr from 0 to 1.0 in steps of 0.1 comparing performance as a function of non‐metabolite backgrounds with the normal human simulation. The nominal FWHM linewidth of 0.05 was held constant. A. full default conditions (dknmtn 0.150) shows a large constant PCr/tCr bias of −0.235 for the native background and wide bias deviations when alternate backgrounds are swapped in. The absence of non‐metabolite background (none) gives a near perfect titration despite FWHM 3 times larger than PCr‐Cre CH_2_ chemical shift separation. B. Small improvements were observed when the standard deviation of individual PCr and Cre shifts allowed by the model were reduced from a default 0.004 to 0.001 ppm (alsdsh) with C. minimal change when adding a MM30 component to the model. D. A setting of dknmtn 0.020 reduced the PCr/Cre bias for the native background, and dramatically reduced bias dependence on alternate backgrounds.

Figure 5 shows titrations of PCr/tCr from 0 to 1 in steps of 0.1. In this comparison FWHM is held constant at 0.050 ppm. A constant PCr/tCr bias for native background is clearly seen with dknmtn set at default 0.150. A wide variation in bias is also observed when swapping in alternate non‐metabolite backgrounds. PCr/tCr bias and the background dependent bias are greatly reduced using dknmtn 0.020. Modest improvements are also realized by reducing the allowable jitter for Cre and PCr alsdsh reduced from default 0.004 to 0.001, plus the addition of an added MM30 component to the 11 presets. It is interesting that the bias appears to remain constant with all slopes near one. With a slope of 1.01, an intercept of 0.005 (*R* ^2^ = 0.9984), the control setup with dknmtn 0.02, aldsdsh 0.001 and added mm30 yielded nearly perfect fit vs. ground truth titrations.

### 3.6 SNR Dependence of Spline

**FIGURE 6:**
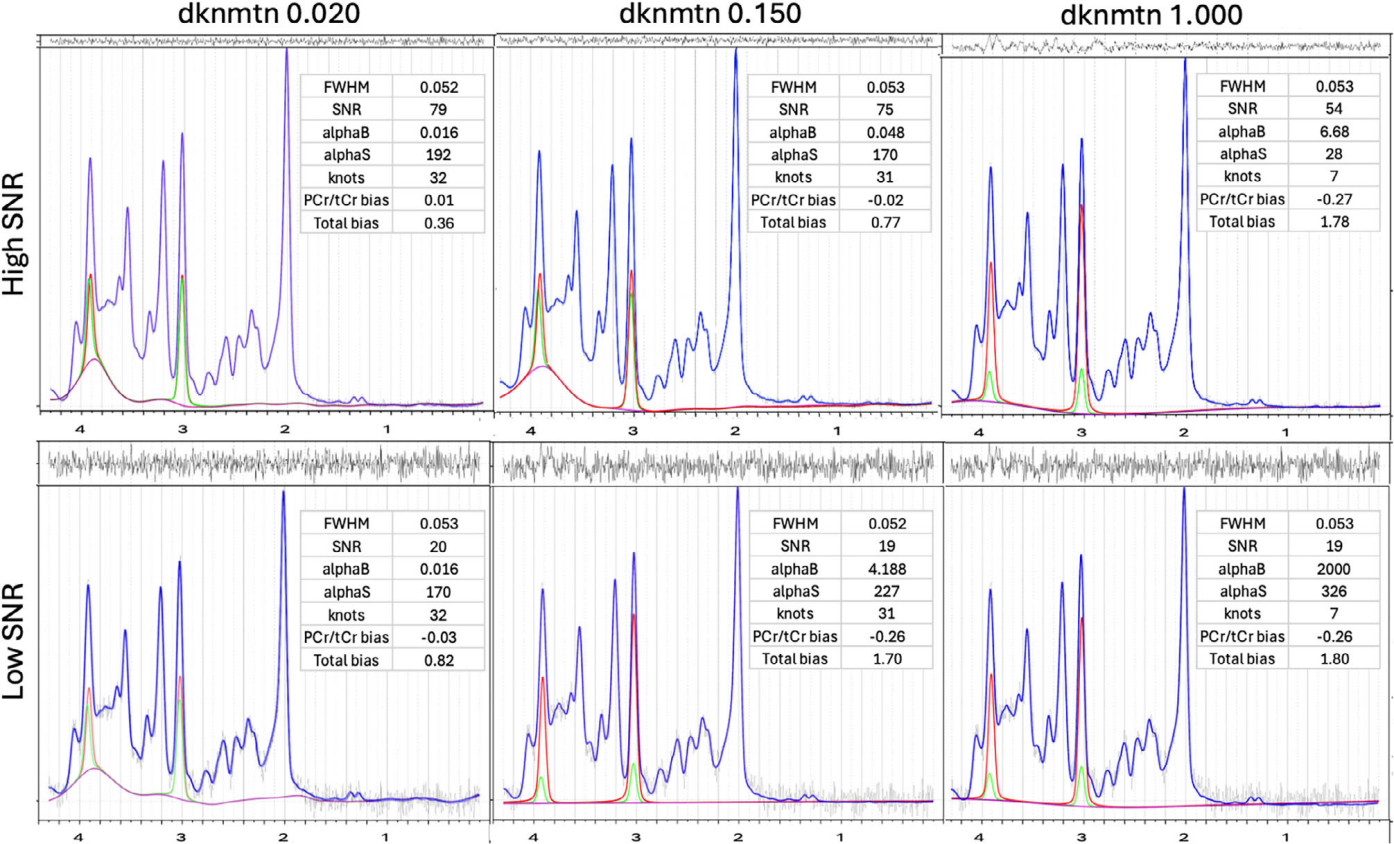
Impact of dknmtn and noise on spline estimates and bias. Overall fit (blue), estimate of 25 Hz Gaussian non‐metabolite background (purple), Cre (red) and PCr (green). Left‐to‐right shows dknmtn 0.020, 0.150 and 1.00. Top row shows high SNR and bottom row lower SNR. The insets provide LCModel outputs for FWHM, SNR, baseline and signal regularization coefficients alphaB and alphaS, along with calculated metrics for PCr/tCr and total biases. A dknmtn setting of 0.020 is best for both high and low SNR, with poor results for default dknmtn of 0.150 at lower SNR and at any SNR with dknmtn set to 1.00.

Figure 6 shows fits at high and low SNR focusing on the spline estimate for the 25 Hz Gaussian background, comparing dknmtn 0.020, 0.150, and 1.00. At default dknmtn 0.150 and lower SNR (20), the smoothing applied by spline regularization prevents an estimate of non‐metabolite background (bottom row, middle). A setting of dknmtn to 1.00 results in a very stiff spline estimate adding bias throughout the spectrum, with poor results even at high SNR.

### 3.7 Asymmetric Lineshape, Alternate Metabolite Content and Bias

**FIGURE 7:**
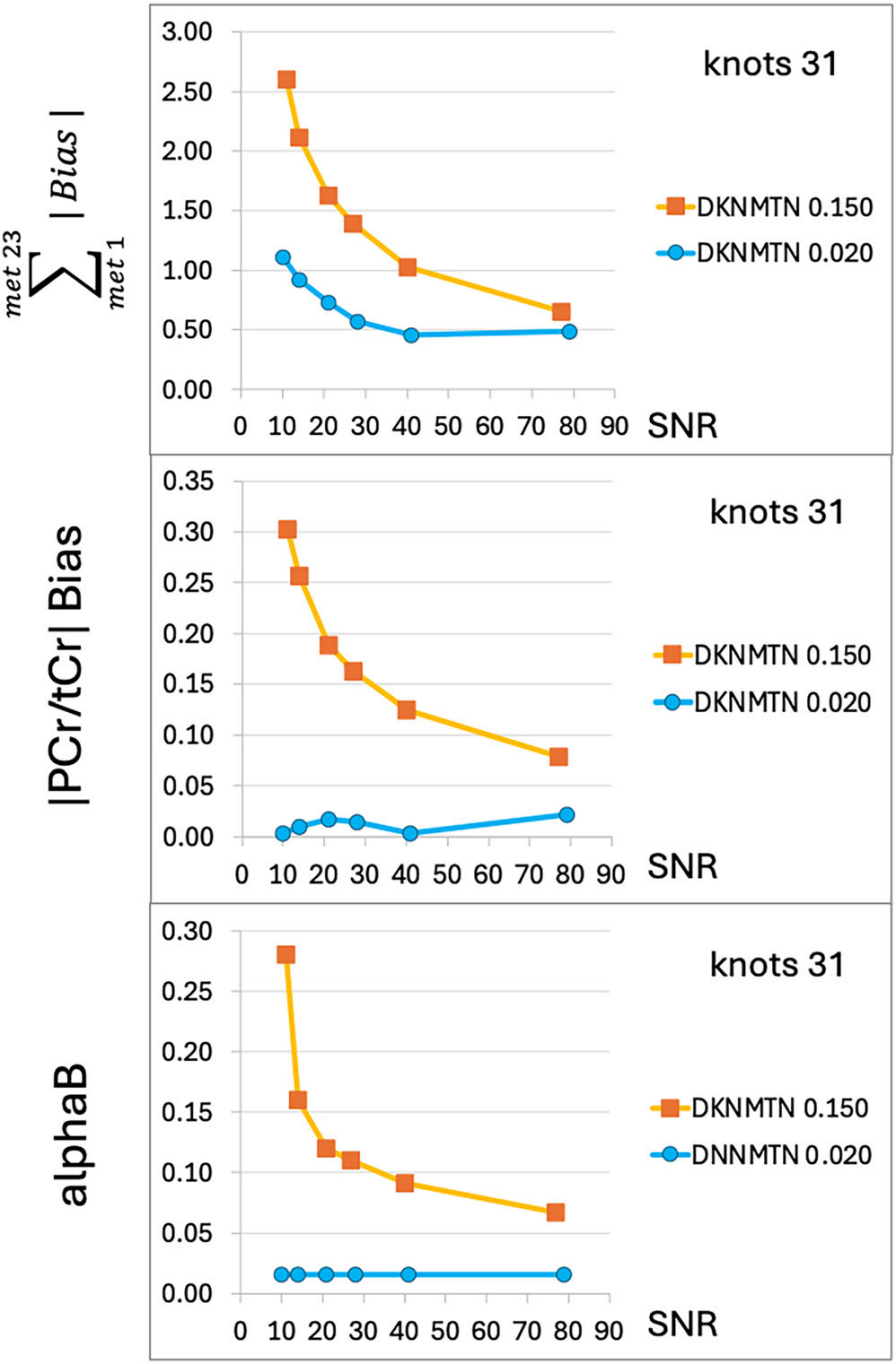
SNR dependence of bias and baseline spline regularization coefficient alphaB. Using the asymmetric lineshape simulation shown in the previous slide, (top) total bias, (middle) absolute value of PCr/tCr bias and (bottom) alphaB are plotted vs. SNR for dknmtn of 0.020 (blue) and 0.150 (orange). Knots are constant.

Although assumed to be separable, regularization of the metabolite signals and non‐metabolite backgrounds is tied in the spline algorithm. To test the effect of added challenges to the metabolite signal regularization problem, an asymmetric lineshape was introduced by shifting 30% of the metabolite signal downfield by 0.02 ppm, and a set of ground truth metabolites from a very different condition (piglet spectrum) was substituted for the normal human set. Comparison of fits using dknmtn 0.020 and default 0.150 is shown in Table 1. These challenges were added to the already difficult conditions of FWHM greater than 0.05 and SNR around 20. Figure 7 shows that although both settings of dknmtn use the same number of spline knots, dknmtn 0.150 shows increasing PCr/tCr bias with decreasing SNR. Although the use of an alternate mixture of metabolites also showed reduced bias with the reduced dknmtn 0.020 setting, the improvement was less dramatic.

**TABLE 1:** Impact of asymmetric lineshape and metabolite mixture on bias.

Lineshape	dknmtn	FWHM	SNR	alphaB	alphaS	knots	PCr/tCr bias	Total bias
Normal	0.150	0.053	22	0.110	340	31	−0.16	1.54
Normal	0.020	0.053	22	0.016	200	33	0.01	0.54
Asymmetric	0.150	0.057	21	0.120	320	31	−0.19	1.63
Asymmetric	0.020	0.057	21	0.016	100	31	0.02	0.73
Asymmetric + PreCA metabolites	0.150	0.057	23	0.150	99	31	−0.14	1.60
Asymmetric + PreCA metabolites	0.020	0.057	23	0.016	410	32	0.08	0.94

*Note*: Starting with a symmetric lineshape of 0.05 Hz FWHM, 30% of metabolite signal was shifted by −0.02 ppm (greater than PCr‐Cre CH_2_ chemical shift separation). The asymmetry increases the effective linewidth estimate, but has minimal effect on bias and the improvement gained with dknmtn 0.020. Swapping in a different set of metabolites from the piglet study shows the same trend with reduced bias at dknmtn 0.020.

### 3.8 dknmtn Setting and the Impact on Total Creatine

**FIGURE 8:**
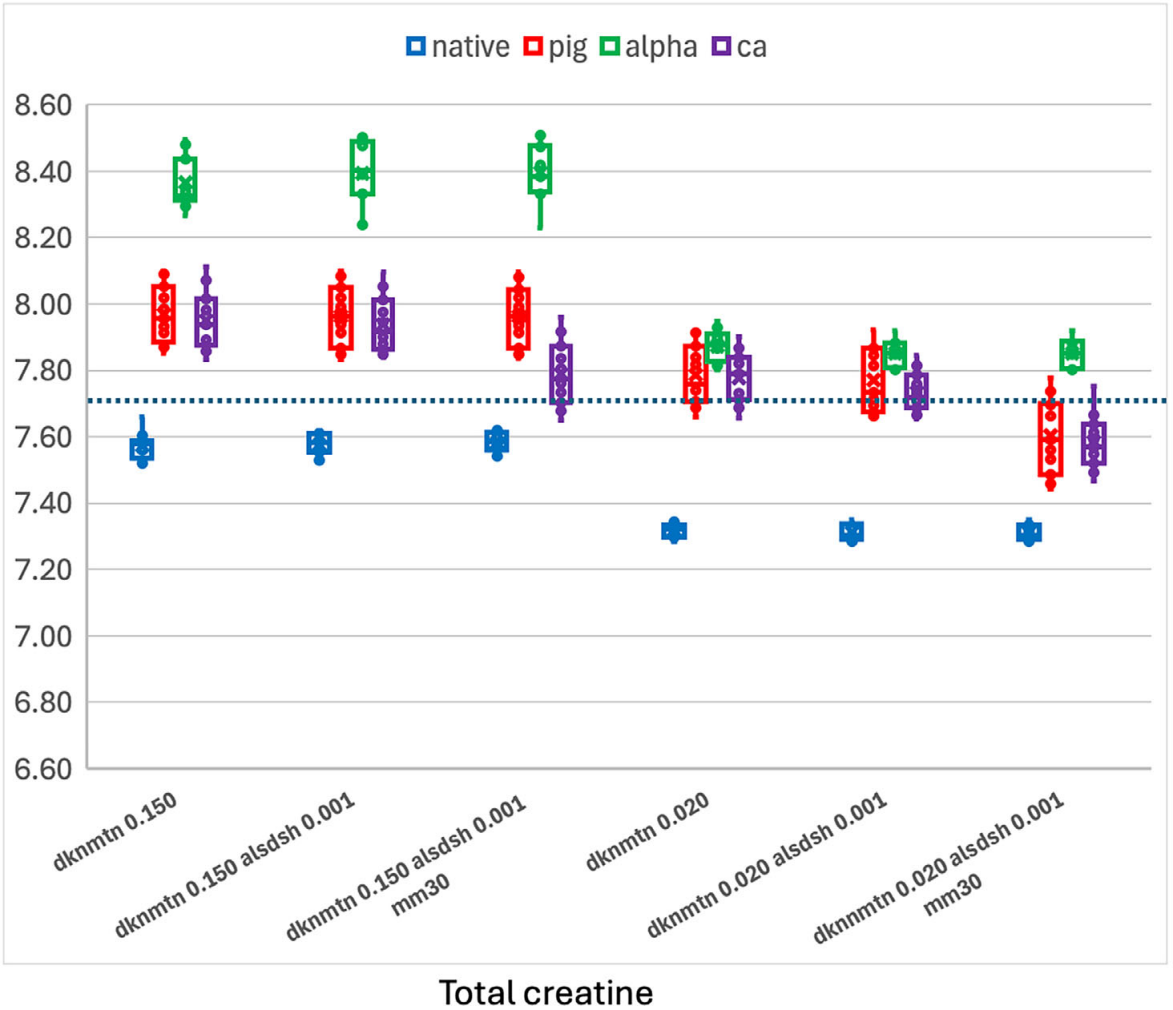
Overestimation and background dependence of total creatine. Background and SNR‐dependent underestimation of non‐metabolite components leads to error in the measurement of total creatine. Modified LCModel setup reduces this added bias.

Incorrect estimation of non‐metabolite signal overlapping Cre and PCr can lead to errors in the estimate of total creatine. This is illustrated in Figure 8 using the lineshape and SNR conditions from Table 1. Errors in total creatine can impact all metabolites that use it as a reference. Some of the reduction in total bias at dknmtn 0.02 may come from a better separation from non‐metabolite signal and therefore a better estimate of the total creatine signal.

### 3.9 PCr Dynamics

**FIGURE 9:**
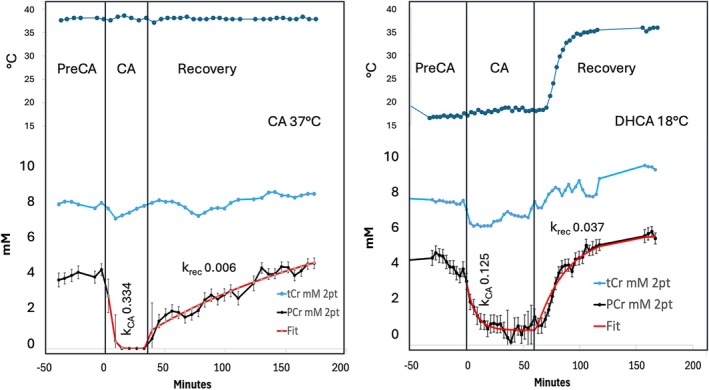
Dynamics of PCr and tCr during two different cardiopulmonary bypass conditions using the modified LCModel setup. On the left pre‐circulatory arrest (PreCA), a 35 min circulatory arrest (CA) and recovery were all done at 37°C reflected in rapid complete loss of PCr *k*_CA_ = 0.334 min^−1^ and a slow recovery of PCr *k*_Rec_ = 0.006 min^−1^ This compares with the DHCA study on the right where PreCA and a 60‐min CA interval are metabolically protected at 18°C. These conditions led to incomplete PCr loss *k*_CA_ = 0.125 min^−1^ and healthier recovery *k*_Rec_ = 0.037 min^−1^ compared to the sever physiological challenge of circulatory arrest at normal body temperature. tCr (blue trace) appears increase during the bypass recovery period, with a negative perturbation possibly at the start of arrest.

Figure 9 shows the dynamics of PCr and tCr (Cre + PCr) estimated with modified LCModel setup in a bypass study in which the temperature remained at 37°C throughout, and in a DHCA study in which the preCA and CA temperatures were maintained at 18°C followed by rewarming and recovery at 37°C. First‐order kinetic fits to the dynamics based on standard LCModel estimates gave a rate constant for the complete loss of PCr during 37°C at *k*_CA_ = 0.334 min^−1^ and the partial loss of PCr during CA at 18°C at *k*_CA_ = 0.125 min^−1^. The relative rates are consistent with biochemical expectations. The recovery from the more damaging 37°C CA conditions is slow *k*_Rec_ = 0.006 min^−1^ compared with recovery from the more protected 18°C CA conditions *k*_Rec_ = 0.037 min^−1^. These results are also consistent with kinetic studies using ^31^P MR spectroscopy. If PCr and Cre are differentiated as shown here, the advantage of the proton approach is the ability to measure the dynamics of both Cre and PCr [18].

### 3.10 Reproducibility and Repeatability

Reducing bias due to non‐metabolite background (Figure 8) and due to SNR and linewidth (Figure 4) should help, but does not guarantee improvement in repeatability or reproducibility. Reproducibility requires testing against larger datasets with multi‐user protocols designed to test this. The dynamic piglet studies provide a limited test of repeatability, with a lack of discontinuity and unchanged CRLBs suggesting no trade‐off in precision for accuracy.

## 4 Discussion

Despite the ^1^H MR spectral similarity of PCr and Cre, LCModel unburdened by non‐metabolite signal, reliably estimates PCr/tCr over a range of SNR (10–100) and linewidths (0.02–0.07 ppm FWHM). Bias is introduced with the addition of non‐metabolite backgrounds. The non‐metabolite background is a combination of macromolecules, out‐of‐band artifact, water and baseline distortion. This background signal can change with species, physiological conditions, location, age, temperature and species. These are not modeled well with default LCModel control parameters and can lead to bias for PCr/tCr and other metabolites. Non‐metabolite signal varies significantly, especially around the 3.9 ppm PCr and Cre CH_2_ resonances and if not estimated well, leads to bias. The non‐metabolite signal below 2.5 ppm is not modeled by default LCModel macromolecule (MM) components and would be difficult to anticipate and include in the basis. As a simple but challenging test, we included a single 25 Hz Gaussian peak as one of the backgrounds. The background observed in piglet circulatory arrest is also potentially challenging as it contains deoxyHb water powder pattern extending into the spectral region of interest. A full range of non‐metabolite backgrounds would be difficult to test, but the four very different contributions in Figure 2 provided a test of background sensitivity as evident in the titration results shown in Figure 5. This leaves estimation of these non‐metabolite and metabolite signals dependent on spline regularization. The regularization of baseline and signal shapes is assumed to be separable which is largely enforced by limiting spline knot spacing to rho*FWHM where rho is defaulted to 2.6 in the version of LCModel tested (6.3‐1 J) [6, 12]. The regularization process can be modified by the control parameter dknmtn. Increase in this parameter over the default 0.150 will increase knot spacing (less knots) leading to a stiffer more constrained baseline as previously explored and reported [13, 19]. Reduction of dknmtn only decreases knot spacing to the rho*FWHM limit. A previous study decreasing dknmtn to 0.05 resulted in an encouraging estimate of the non‐metabolite signal without any prior knowledge [2]. The only limitation appeared to be excess smoothing of the narrowest macromolecule signals. In this study we explored lower values of dknmtn to find an optimum value based on bias. Even in the absence of changes in knot spacing, dknmtn appears to impact spline smoothing via the baseline regularization coefficient alphaB. At dknmtn values of 0.03 or higher, alphaB, the smoothing of the background spline and bias increases at lower SNR. As illustrated in Figures 6 and 7, SNR impacts the spline estimate and bias in the measurement of PCr/tCr. Optimizing LCModel control parameters based on both PCr/tCr and total metabolite bias helps test the assumption that the rho*FWHM limit avoids metabolite errors due to overfitting the non‐metabolite background. The optimum setting for dknmtn 0.020 reduces bias in the measurement of PCr/tCr independent of non‐metabolite background, SNR and lineshape over the ranges tested. Increased knot spacing at higher FWHM linewidths than tested is expected to reduce performance of baseline regularization and increase bias.

Additional minor (improvements) in the LCModel control parameters include a reduction in the standard deviation allowed for the individual shifts of Cre and PCr setting alsdsh to 0.001, down from the default of 0.004 ppm. These values are below the (0.005 ppm) digital resolution of the sampling used but seemed to help. Likewise a possible improvement was observed with the addition of MM30 an additional LCModel macromolecule component. Although LCModel with the default setup was sufficient to see a correlation between apparent PCr/tCr and NAA loss during circulatory arrest [5], validation and improvements are important to extend the metric to the kinetics of the PCr changes. PCr/tCr dynamics yielding plausible kinetics in cardiopulmonary bypass studies run under different conditions were demonstrated. The improvements beyond more accurate differentiation of Cre and PCr, measured in this study as the sum of the absolute biases for all metabolites are interesting. In future studies we will explore the impact of the modified LCModel setup on key individual metabolite metrics like Gln/Glu [8] and Suc [9] that we have previously correlated with bypass conditions.

Limitations of the current study include low out‐of‐band and low system‐level baseline distortion in the tested non‐metabolite backgrounds. A very small set of different ground truth metabolite combinations, and only modest metabolite peak asymmetry in this study are potentially important limits. The less dramatic reduction in PCr/tCr bias (default dknmtn 0.150 to 0.020) on switching the mixture of metabolites in Table 1, suggests that bias is not only dependent on the separation of metabolite from non‐metabolite, but is also dependent on separating metabolite from metabolite. More extensive testing is likely required to understand the interdependence of metabolites that partially overlap the PCr and Cre CH_2_ resonances (i.e., Asp, Asc, Glc, PE, and GPC). Linewidths over 0.07 ppm were also avoided and expected to perform poorly due to a minimum knot spacing over 0.180 ppm. Also no attempt was made toward including an experimental macromolecule contribution into the basis set. Both simulations and fitting used a set of largely experimental 23‐metabolite basis spectra. More general use of the modified setup would require testing with different basis set choices, including the experimental macromolecule component if appropriate. Retrospective analysis of other preclinical and clinical MRS findings would also help determine general value. Before adopting the control parameters provided in Table S2 as a best practice, testing repeatability and reproducibility with existing or prospective data is needed. For future testing it would also be valuable if the estimates of non‐metabolites and metabolites could be directly output from the model. This would eliminate the need for subtraction in the present simulation protocol, and with improved estimates, provide a set of more challenging backgrounds.

## 5 Conclusions

Setting the LCModel control parameter dknmtn to 0.020 reduces PCr/tCr bias. This setting also reduces overall metabolite bias. Setting dknmtn above the default 0.150 should only be used with high confidence that the remaining baseline is very constrained. Under modified setup conditions, excellent fit results are demonstrated for PCr/tCr over a range of SNR, lineshape, non‐metabolite backgrounds. Excellent linear fit correlations in simulated PCr titrations add confidence for using proton MRS to study PCr dynamics.

## Conflicts of Interest

The authors declare no conflicts of interest.

## Supporting information


**Table S1:** MRSinMRS.
**Table S2:** Modified LCModel control parameters.
**Table S3:** PCr and Cre Chemical Shifts.
**Figure S1:** Temperature dependence of PCr and Cre chemical shifts.
**Figure S2:** Spline bias at high dknmtn values.
**Figure S3:** Reduction of bias beyond PCr/tCr.
**Figure S4:** Simulation schematic.
**Figure S5:** Simulation matrix flow diagram.
