# Supplementary material for: Differentiating Creatine and Phosphocreatine In Vivo Using 3 T 1H MR Spectroscopy
Source: Magn Reson Med. 2025 Nov 9;95(4):1896–906. doi: 10.1002/mrm.70171 (PMC12850560; doi:10.1002/mrm.70171)
Supplement: Supplementary file 1 — Table S1: MRSinMRS. Table S2: Modified LCModel control parameters. Table S3: PCr and Cre Chemical Shifts. Figure S1: Temperature dependence of PCr and Cre chemical shifts. Figure S2: Spline bias at high dknmtn values. Figure S3: Reduction of bias beyond PCr/tCr. Figure S4: Simulation schematic. Figure S5: Simulation matrix flow diagram. [file MRM-95-1896-s001.pdf]

Received: 00 Month 0000 | Revised: 00 Month 0000 | Accepted: 00 Month 0000
DOI: xxx/xxxx

Differentiating Creatine and Phosphocreatine In Vivo Using 3T ^{1}H MR Spectroscopy. Supplemental Material

Ralph E. Hurd[1] | Meng Gu[1] | Kenichi Okamura[1] | Masafumi Shibata[1] | Yoshikazu Ono[1] | Moussa Haidar[1] | R. Kirk Riemer[1] | Frank L. Hanley[1] | Daniel M. Spielman[1]

Supplemental Material:

Table S1. MRSinMRS:
Table S2. Modified LCModel control parameters:
Table S3. PCr and Cre Chemical Shifts:
Figure S1. Temperature dependence of PCr and Cre chemical shifts:
Figure S2. Spline bias at high dknmtn values:
Figure S3. Reduction of bias beyond PCr/tCr:
Figure S4. Simulation schematic:
Figure S5. Simulation matrix flow diagram:

Table S1. MRSinMRS Table.

Site (Stanford)<br><br>1. Hardware			
a. Field strength [T]	3T	3T	3T
b. Manufacturer	GE Healthcare	GE Healthcare	GE Healthcare
c. Model (software version if available)	UHP (MR30.1)	MR750 (MR29)	MR750 (MR27)
d. RF coils: nuclei (transmit/ receive), number of channels, type, body part	Body Transmit<br><br>32 ch Nova Medical head coil receive	Body Transmit<br><br>16 channel flex receive (GEM Flex Medium)	Body Transmit<br><br>8 channel knee coil
e. Additional hardware			
2. Acquisition			
a. Pulse sequence	sLASER w/focal mrsi	sLASER w/sensitive point filter (SPF)	sLASER
b. Volume of Interest (VOI) locations	50 x 30 x 10 mm^3 sum of 5x3 1cc subvoxels within focal excited volume of 79 x 59 x 10 mm^3	12 x 12 x 15 mm^3 right midbrain (contains cortical and sub-cortical)	12 x 12 x 15 mm^3 right midbrain (contains cortical and sub-cortical)
c. Nominal VOI size [cm^3, mm^3]	15 cm^3	2.1 cm^3	2.1 cm^3
d. Repetition Time (TR), Echo Time (TE) [ms, s]	TR/TE 2000/30	TR/TE 2000/30	TR/TE 2000/30
e. Total number of Excitations or acquisitions per spectrum<br><br>In time series for kinetic studies<br><br>i. Number of Averaged spectra (NA) per time-point<br>ii. Averaging method (e.g. block-wise or moving average)<br>iii. Total number of spectra (acquired / in time-series)	NA = 1 (16 x 16 FOV 160 2D mrsi)	NA = 64<br><br>i. 2<br><br>ii. 2 Pt-Moving average<br><br>iii. 52 (26) interleaved	NA = 128<br><br>i. 2<br><br>ii. 2 Pt-Moving average<br><br>iii. 25

f. Additional sequence parameters<br><br>(spectral width in Hz, number of spectral points, frequency offsets)<br><br>If STEAM:, Mixing Time (TM)<br><br>If MRSI: 2D or 3D, FOV in all directions, matrix size, acceleration factors, sampling method	Spectral Width 5000Hz 4k complex points	Spectra Width 5000Hz 4k complex points<br><br>SPF = 2D MRSI 8x8 FOV 120 mm^2<br><br>(Filter 15 x 15 mm^2)	Spectral Width 5000Hz 4k complex points
g. Water Suppression Method	VAPOR with 5% residual water by design	VAPOR with 5% residual water by design	VAPOR with 5% residual water by design
h. Shimming Method, reference peak, and thresholds for “acceptance of shim” chosen	Linear x,y,z. second order+z3<br><br><8Hz water	Linear x,y,z water<br><br><8Hz water	Linear x,y,z water<br><br><8Hz water
i. Triggering or motion correction method<br><br>(respiratory, peripheral, cardiac triggering, incl. device used and delays)	Frame by frame ECC on residual water	Frame by frame ECC on residual water	Frame by frame ECC on residual water
**3. Data analysis methods and outputs**			
a. Analysis software	SAGE, Matlab, LCModel 6.3-1J	SAGE, Matlab, LCModel 6.3-1j	SAGE, Matlab, LCModel 6.3-1J
b. Processing steps deviating from quoted reference or product	restricted-band eddy current correction[16].	pure water subtraction with restricted-band eddy current correction[16].	pure water subtraction with restricted-band eddy current correction[16].
c. Output measure<br><br>(e.g. absolute concentration, institutional units, ratio)<br>Processing steps deviating from quoted reference or product	ratios to tCr	Estimated absolute from ratios to tCr	Estimated absolute from ratios to tCr

d. Quantification references and assumptions, fitting model assumptions			Assume tCr = 8 mM
4. Data Quality			
a. Reported variables<br><br>(SNR, Linewidth (with reference peaks))	*SNR 38:1*<br><br>*LW 0.024 ppm*	*SNR 25:1*<br><br>*LW 0.024 ppm*	*SNR 21:1*<br><br>*LW 0.024 ppm*
b. Data exclusion criteria	*none*	*none*	*none*
c. Quality measures of postprocessing Model fitting (e.g. CRLB, goodness of fit, SD of residual)	*Simulated spectra using experimental non-metabolite signal used for validation*	*Simulated spectra using experimental non-metabolite signal used for validation*	*Simulated spectra using experimental non-metabolite signal used for validation*
d. Sample Spectra	A. MRSinMRS_1	B. MRSinMRS_2	C. MRSinMRS_3

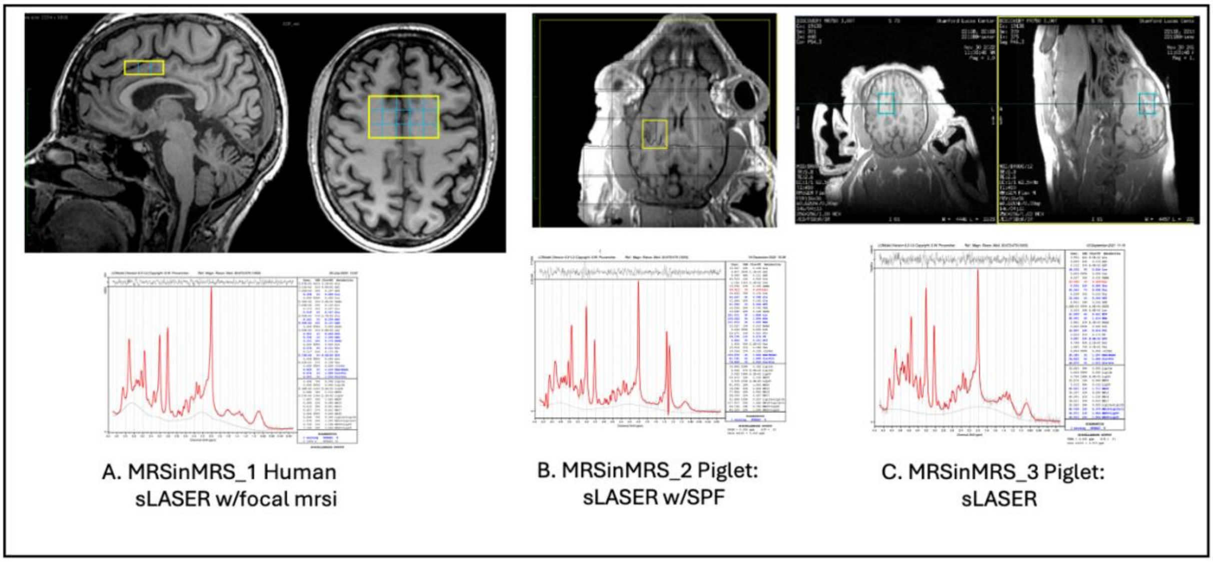

TABLE S2 Modified LCModel Control File

```
NSIMUL=14
CHSIMU(14)='MM30=3.0+-.02 FWHM=.14 <.17+-0.15 AMP=3'
CHRATO(14) = 'MM30/MM09= 0.5 +- 0.55'
NSDSH=2
CHSDSH(1)='Cre'
CHSDSH(2)='PCr'
ALSDSH(1)=0.001
ALSDSH(2)=0.001
DKNMTN = 0.02
sddegz=3.0
sddegp=3.0
PPMST= 4.3
PPMEND= 0.1
NORATO(1)=Glc
```

TABLE S3 PCr and Cre chemical shifts at 37°C and temperature dependence used in basis spectra

Metabolite	Moiety	ppm
PCr	CH_2	3.930
PCr	CH_3	3.031
Cre	CH_2	3.913
Cre	CH_3	3.026
PCr-Cre	CH_2	0.017
PCr-Cre	CH_3	0.005
PCr ppm/°C	CH_2	-0.00044
PCr ppm/°C	CH_3	0.00023
Cre ppm/°C	CH_2	-0.00040
Cre ppm/°C	CH_3	0.00020

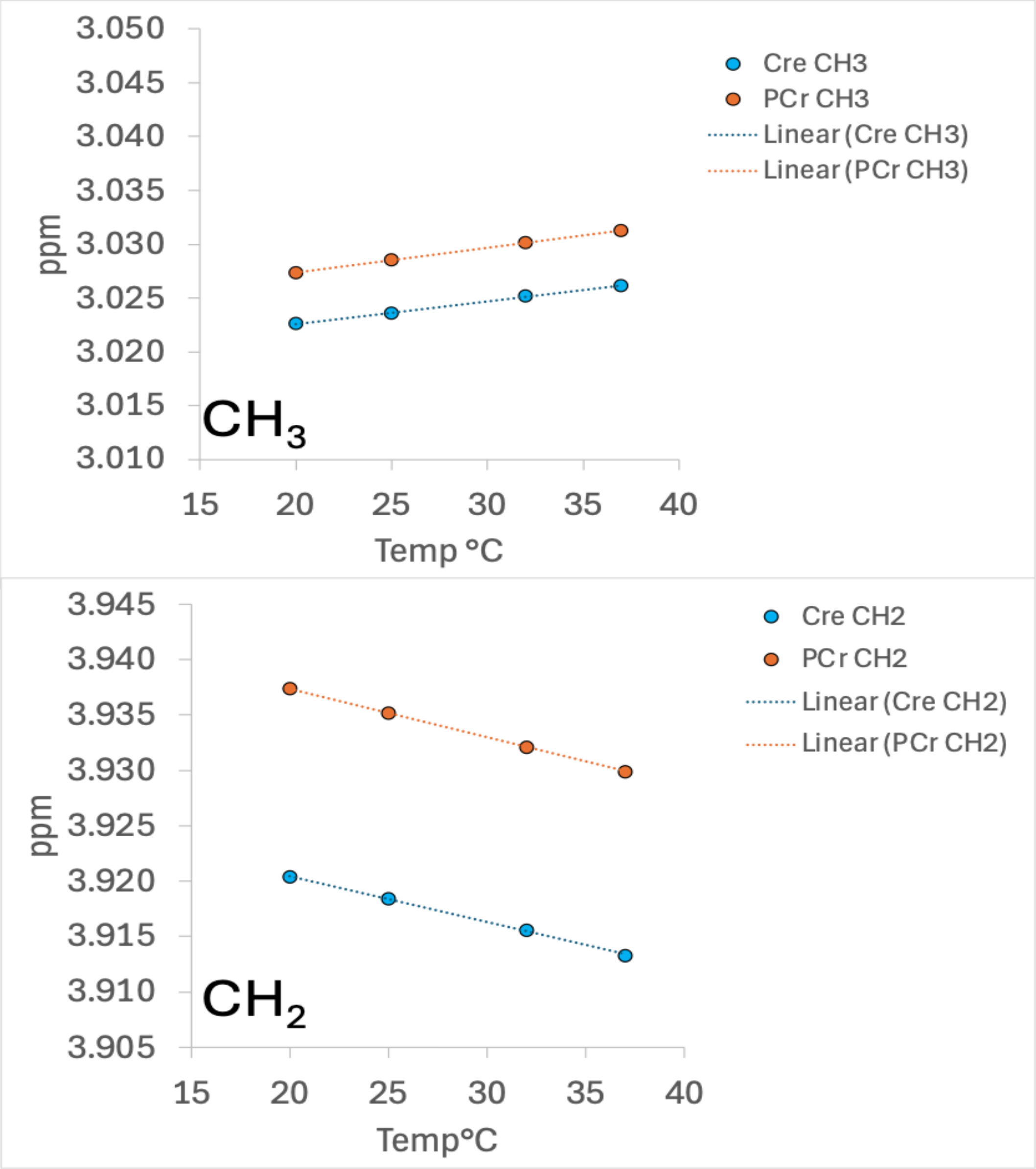

FIGURE S1 Temperature dependence of PCr and Cre chemical shifts

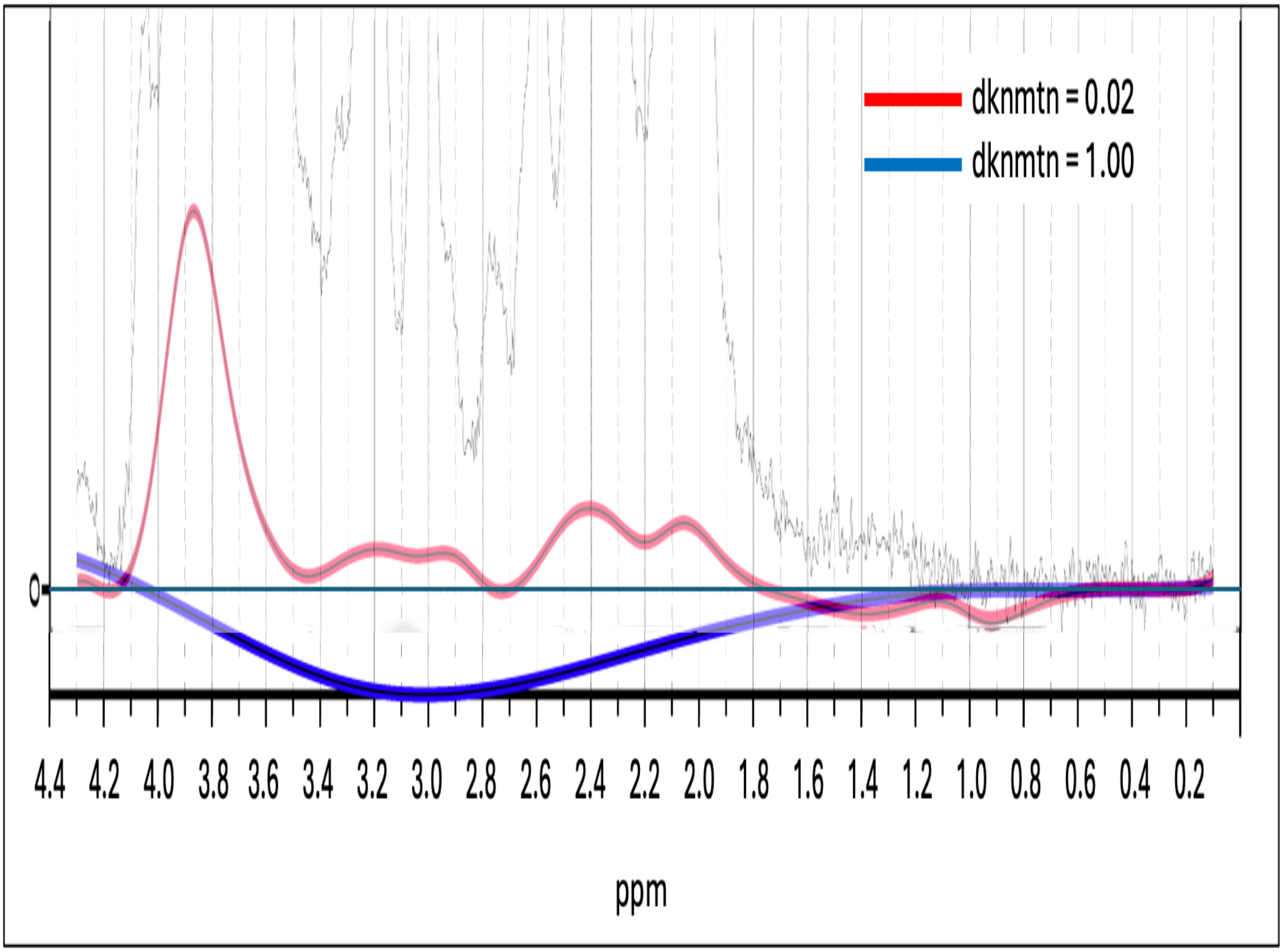

FIGURE S2 Spline baseline error at high dknmtn values. In the absence of prior knowledge, a heavily constrained spline at dknmtn = 1.00 introduces baseline dependent bias beyond absence of baseline fit. The assumption for the use of very stiff spline parameters seems to be the inclusion of a near ideal non-metabolite background in the basis set. In an instance where the non-metabolite background is primarily macromolecule signal, and where the macromolecule signal has been faithfully determined experimentally, a limited spline may help. Otherwise, selection of high dknmtn values is not recommended.

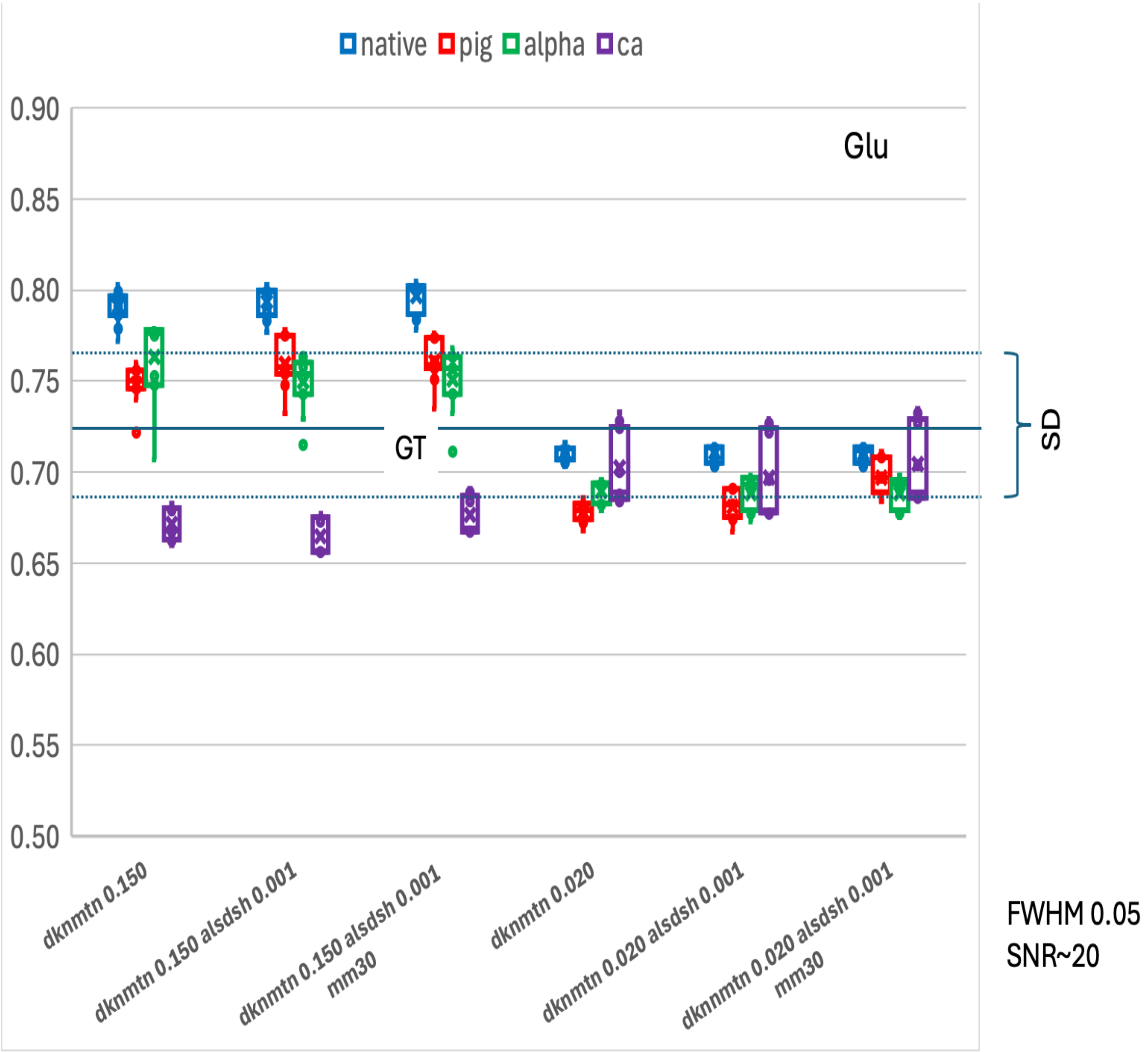

FIGURE S3 Evidence for reduction in bias for individual metabolites beyond PCr/tCr. Glu/tCr example.

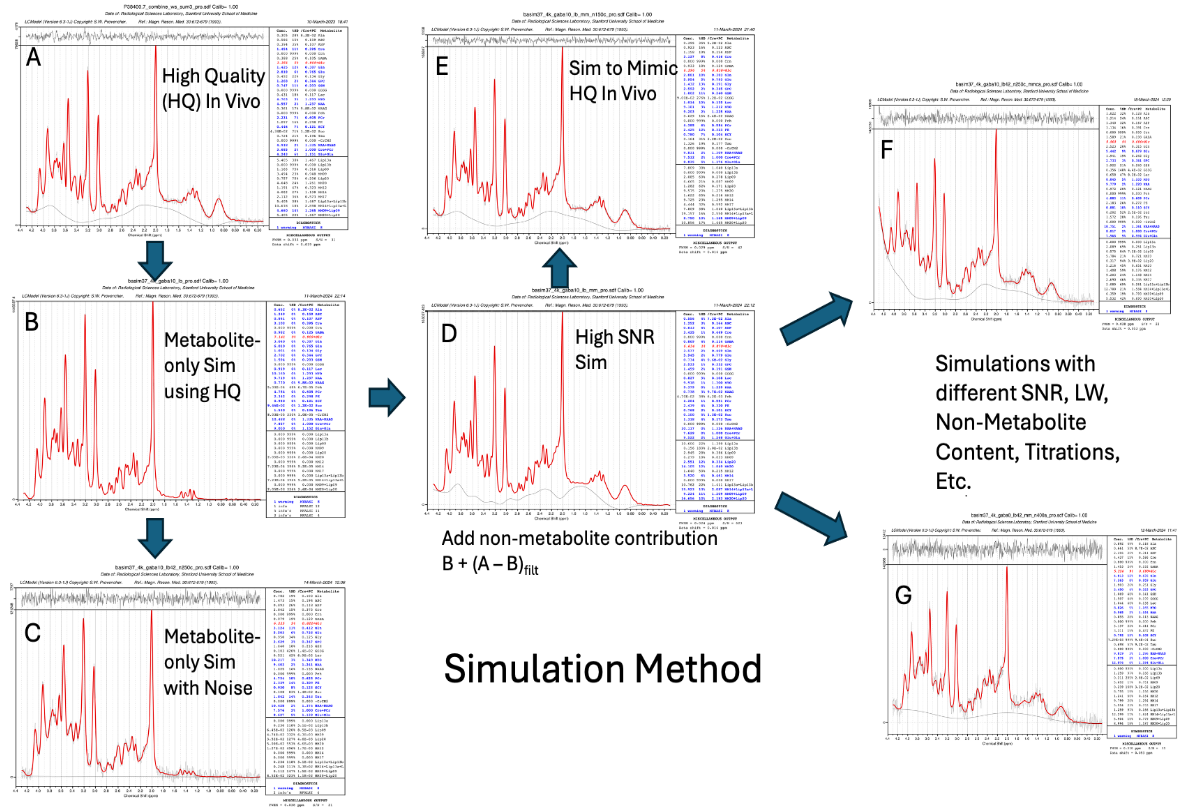

FIGURE S4 Schematic for Simulations

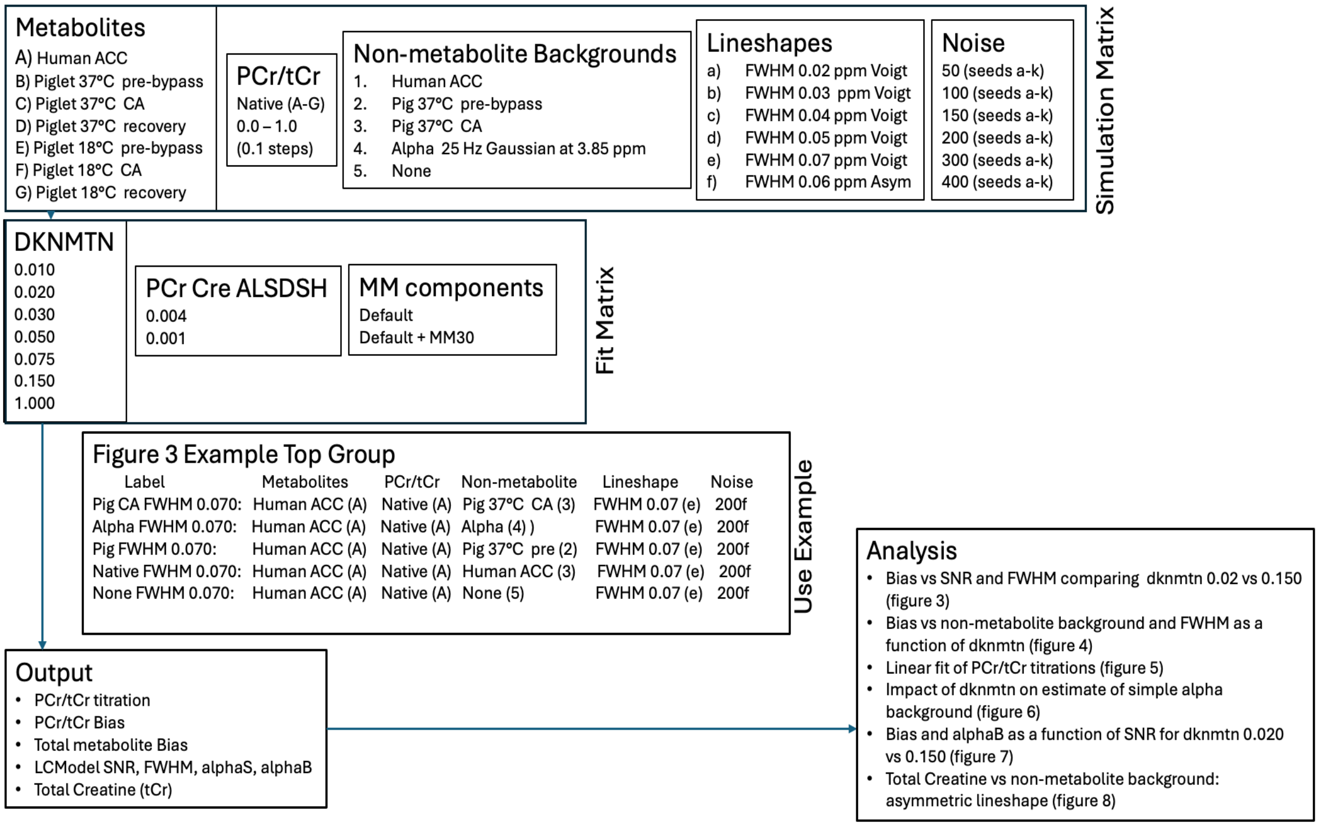

FIGURE S5 Simulation Matrix Flow diagram
